# A Rare Case of Urinary Bladder Exstrophy Presenting With Signet Ring Cell Adenocarcinoma in an Adult

**DOI:** 10.7759/cureus.34308

**Published:** 2023-01-28

**Authors:** Gaurav Kumar Malvi, Sujata K Patwardhan, Bhushan Patil, Rajvi Goradia, Abhinav Malvi

**Affiliations:** 1 Urology, King Edward Memorial (KEM) Hospital and Seth Gordhandas Sunderdas (GS) Medical College, Mumbai, IND; 2 Surgery, Jawaharlal Nehru Medical College, Datta Meghe Institute of Higher Education and Research, Wardha, IND

**Keywords:** pedicled flap, ileal conduit, signet ring cell adenocarcinoma, cystectomy, urinary bladder exstrophy

## Abstract

Due to the conspicuous morphology of the deformity and the fact that primary reconstruction is typically performed in infancy, untreated bladder exstrophy in adults is infrequent. An adult presenting with bladder exstrophy is quite uncommon. We present a 32-year-old man with a bladder mass that existed since birth. He complained of an unpleasant discharge from the mass upon presentation, and on examination, a mass was seen on the urinary bladder's exposed surface, coupled with penile epispadias, a deformed scrotum, and undersized bilateral testicles. Ultrasonography of the kidneys, ureters and urinary bladder (USG KUB), contrast-enhanced computed tomography (CECT) of the abdomen and pelvis, and mass biopsy were all used to investigate the patient. The patient was found to have signet ring adenocarcinoma of the urinary bladder. A radical cystectomy with an anterolateral thigh flap was performed. The clinical and radiological characteristics, treatments, and results of this uncommon presentation are discussed in this case report.

## Introduction

Bladder exstrophy is uncommon, occurring 2.15 times per 100,000 live births [1]. Nearly all patients receive therapy immediately after delivery since the abnormality has a visible morphology at birth. Numerous people are nonetheless at risk for developing cancer, according to studies [2]. Only about 90 primary cancer cases of untreated bladder exstrophy have been reported in the literature. Only two of them were signet ring cell mucinous adenocarcinomas [3, 4].

We present the third case of signet ring cell adenocarcinoma in a 32-year-old man with untreated bladder exstrophy to highlight the extreme rarity, though a distinct possibility, and difficulties encountered in the surgical treatment of such patients.

## Case presentation

**Figure 1:**
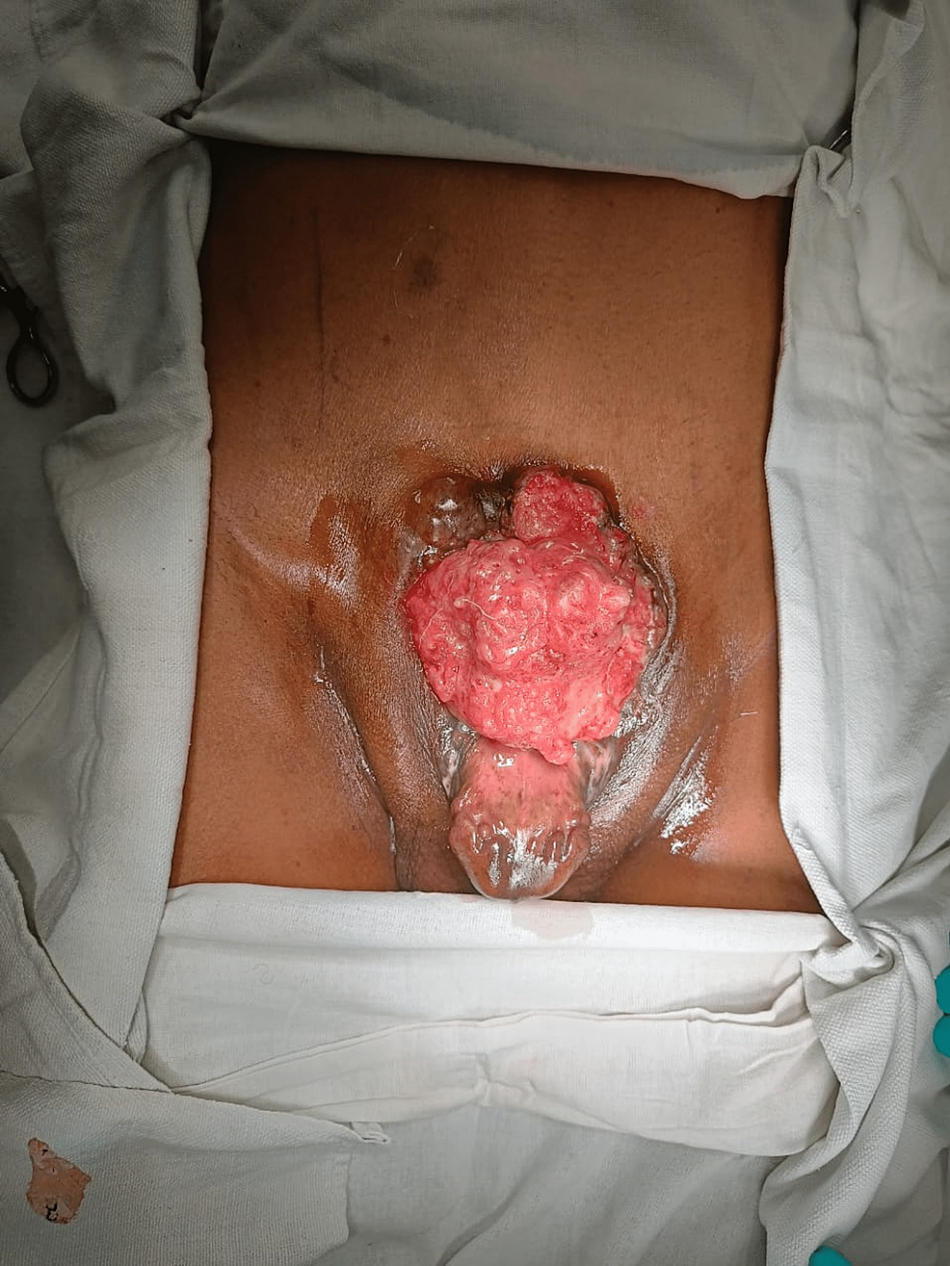
A clinical photograph showing the bladder mass

A 32-year-old unmarried man had had a bladder mass since birth. He had never consulted a doctor for the appearance of a lower abdominal mass and now presented with a foul-smelling discharge from the mass. On examination, there is an approximately 12 cm × 10 cm sized, an exophytic irregular mass that was non-tender and bled on touch and occupies the entire exposed urinary bladder surface (Figure 1). Penile epispadias was present. His scrotum was underdeveloped, with bilateral small testes. Bilateral inguinal lymph nodes were enlarged and firm in consistency.

The patient had a history of acute kidney failure before he presented to us with a serum creatinine of 7.5 mg/dl. An ultrasound of the abdomen and pelvis was done, which was suggestive of bilateral moderate hydroureteronephrosis, for which bilateral percutaneous nephrostomy tube insertion was done, following which the creatinine decreased up to 1.5 mg/dl. Blood investigations revealed hemoglobin of 10.1 g/dl and serum bilirubin of 0.6 mg/dl.

**Figure 2:**
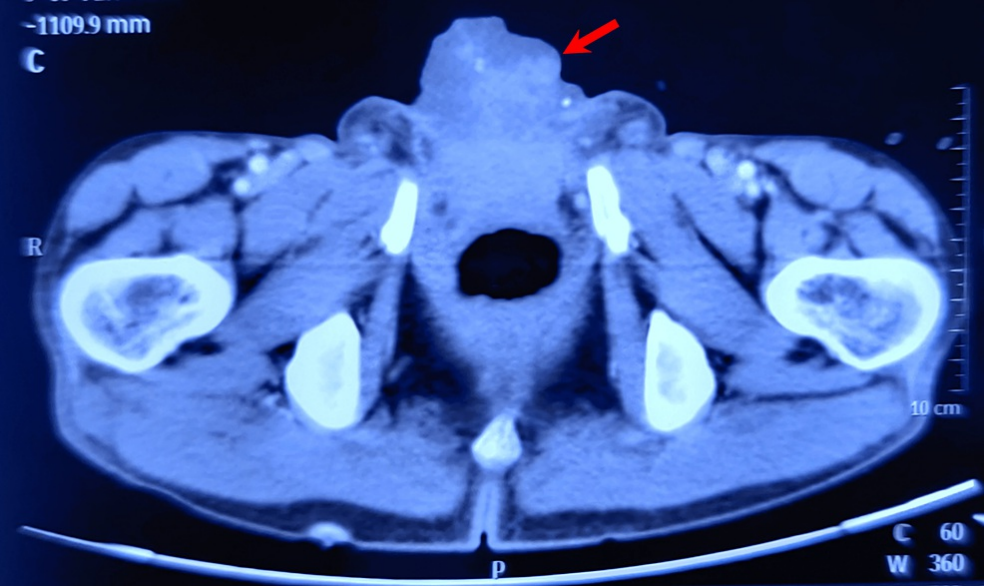
A CT scan showing the mass arising from bladder exstrophy

Contrast-enhanced computed tomography of the abdomen and pelvis revealed a soft tissue mass protruding from a large inferior abdominal wall lesion of size 5.3 cm with widened pubic symphysis and an absent urinary bladder, consistent with bladder exstrophy (Figure 2). The computed tomography (CT) of the chest was normal. The fine-needle aspiration cytology of bilateral inguinal nodes did not show any evidence of malignancy.

**Figure 3:**
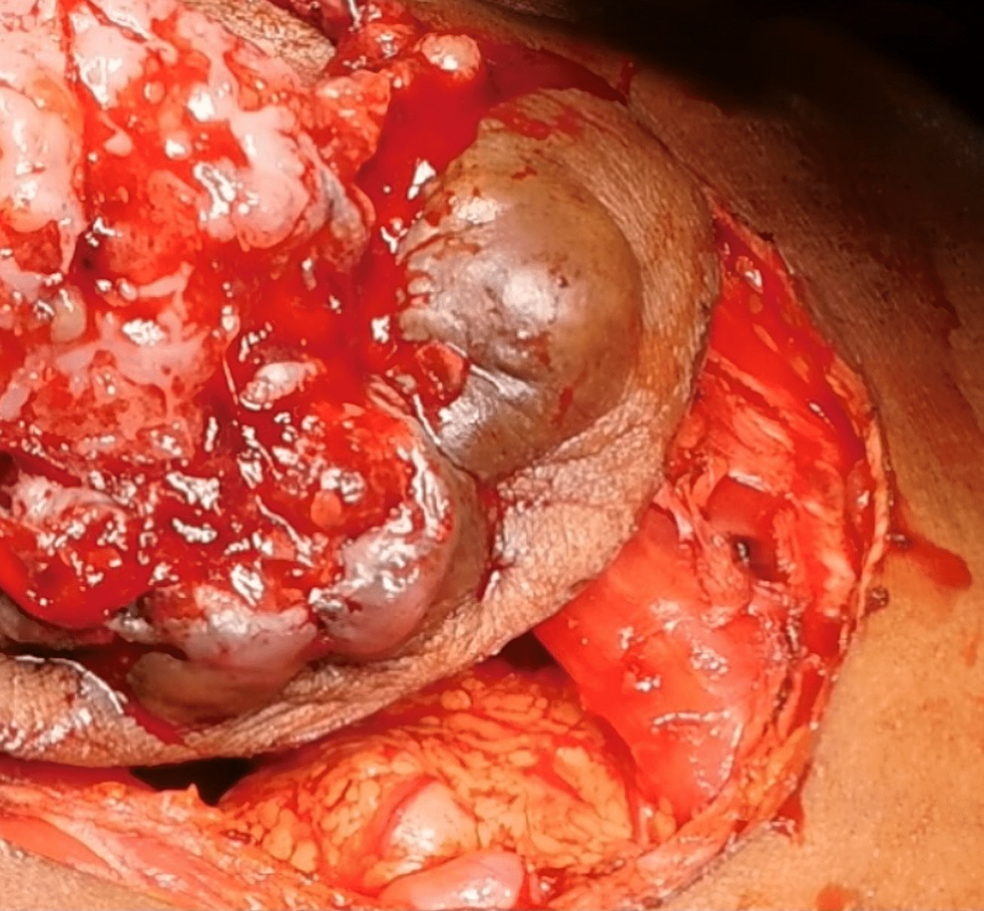
Wide excision of the bladder mass with a 1 cm margin

**Figure 4:**
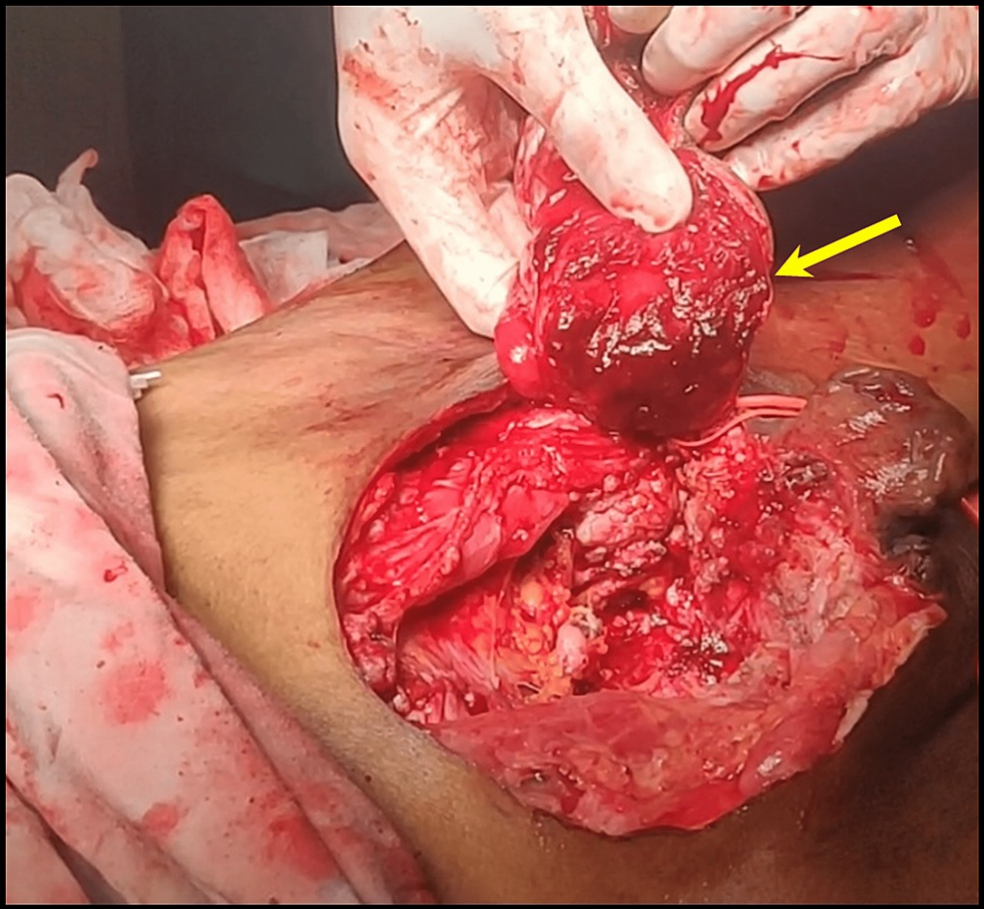
Left anterolateral thigh flap (arrow)

**Figure 5:**
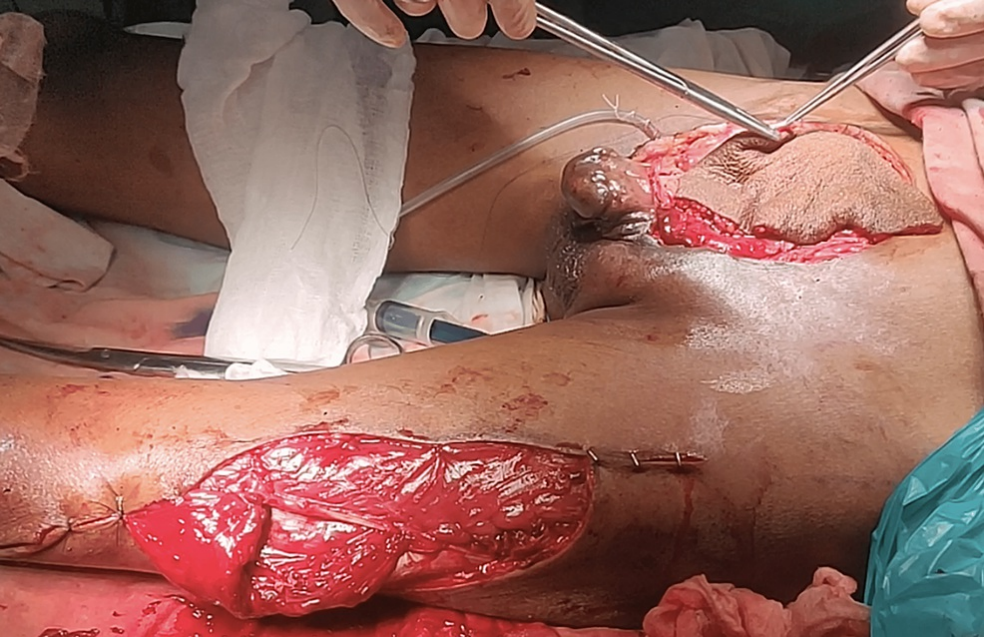
The defect is covered with a pedicle of the anterolateral thigh flap based on perforators of the descending branches of the lateral circumflex femoral artery

Subsequently, a radical cystectomy was carried out with wide local skin excision with a 1 cm margin of resection (Figure 3), along with an ileal conduit. After a significant local resection, the abdominal defect (Figure 4) of approximately 13 cm x 11 cm was covered with a pedicle of the anterolateral thigh flap based on perforators of descending branches of the lateral circumflex femoral artery (Figure 5).

**Figure 6:**
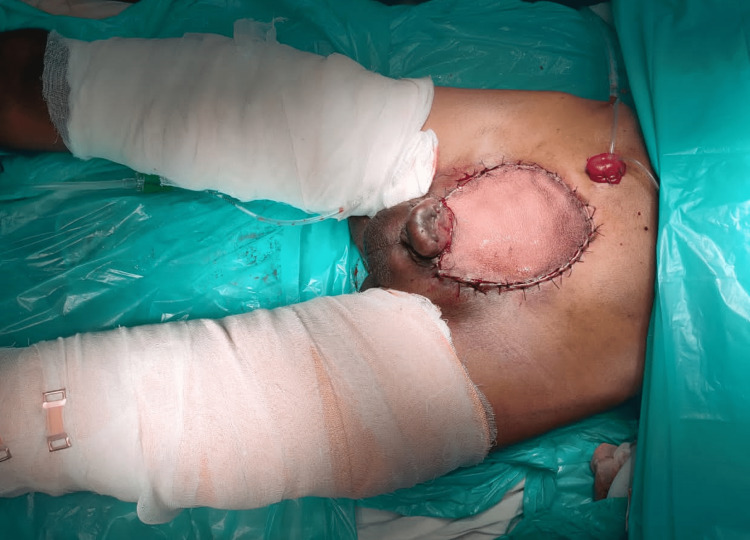
The defect site covered after the surgery

**Figure 7:**
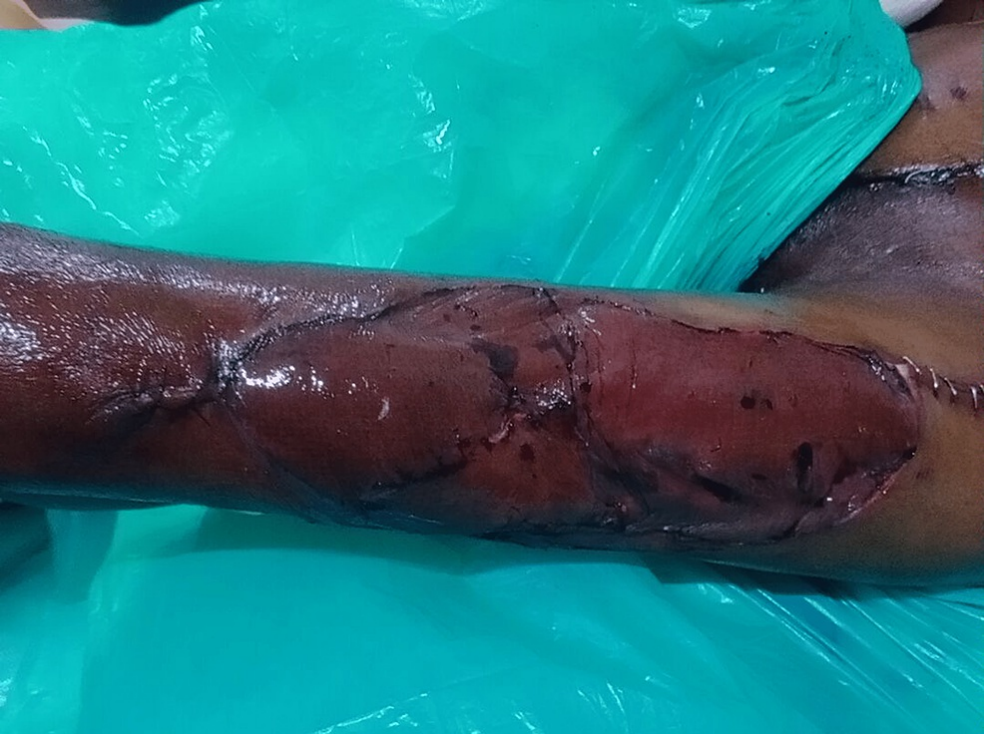
The flap site after the surgery

The defect (Figure 6) and flap (Figure 7) sites appeared healthy post-completion of surgery.

The postoperative period was unremarkable.

**Figure 8:**
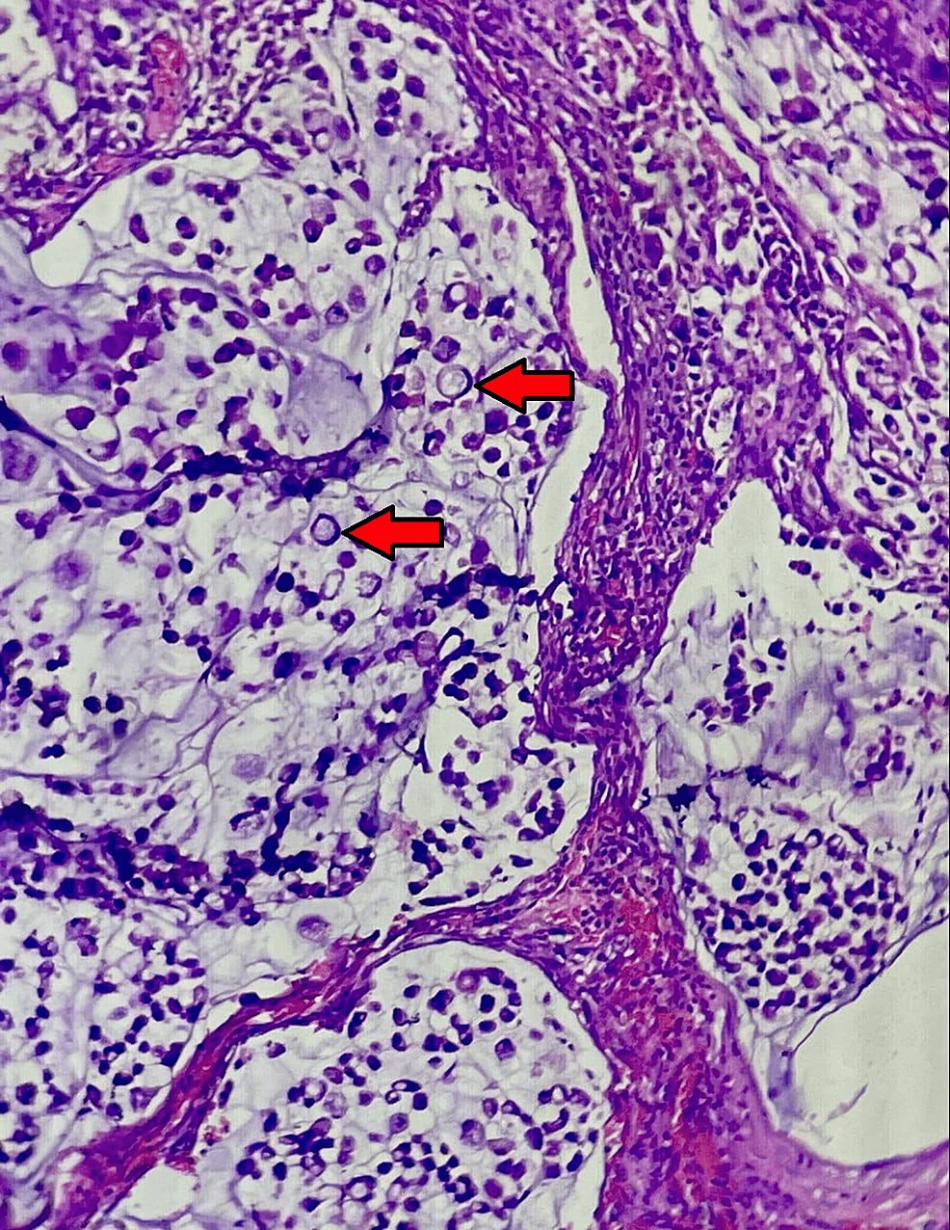
Histopathological (hematoxylin and eosin stain) examination of the resected mass (tumor) at low power magnification (10X) (arrow)

**Figure 9:**
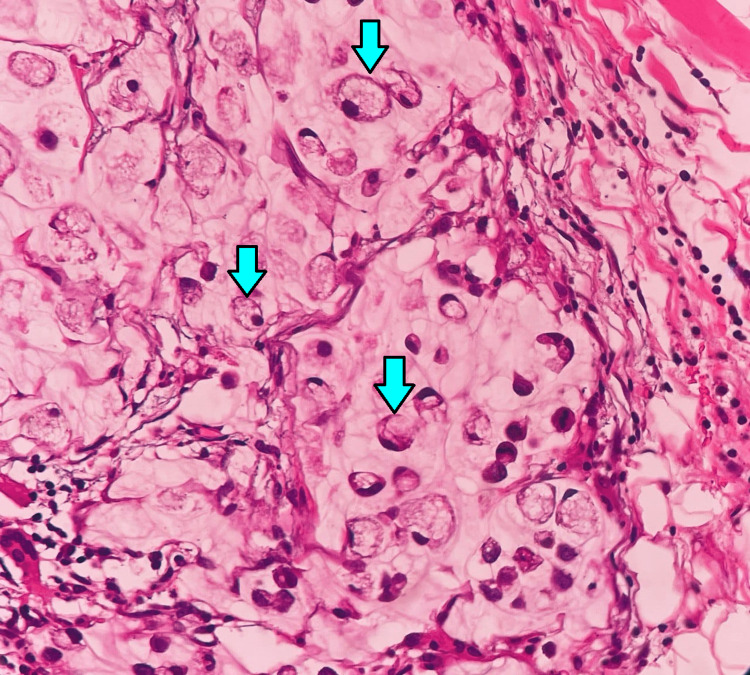
Histopathological (hematoxylin and eosin stain) examination of the resected mass (tumor) on high power magnification (40X) (arrow)

The specimen of the bladder mass was sent for histopathological examination (HPE), which was suggestive of signet ring cell adenocarcinoma under low (Figure 8) and high power magnification (Figure 9). The ureteric and prostatic margins were free of tumors. The HPE of the left inguinal node excised during harvesting of the pedicle of the anterolateral thigh flap was suggestive of tumor deposits of signet ring cell adenocarcinoma as well as extra-nodular tumor deposits.

## Discussion

Bladder exstrophy is a rare disorder with an incidence of 2.15 per 100,000 live births [1]. Since the defect is so obvious, almost all patients undergo treatment soon after birth. Studies show that even after reconstruction, many people are still susceptible to cancer. According to Smeulders et al., neoplasia manifests in 17.5% of treated patients with exstrophy. A bladder neoplasia risk of 4% was present in patients with treatment-resistant exstrophy, which was 700 times higher than the risk in the general population [2]. It is far more unusual for someone to have bladder exstrophy beyond age 50. Both developed and developing countries have reported incidents of this nature. They are probably reluctant to seek medical attention because of social neglect and a lack of support.

Only a very small portion of patients had bladder cancer that had not spread. A thorough literature search turned up fewer than 90 occurrences of primary bladder cancer in persons with untreated exstrophy. There are just two of these occurrences that are known to have their roots in India [3, 4].

The possibility of developing cancer from untreated bladder exstrophy was originally identified in 1955 by McIntosh and Worley [2,5]. Two-thirds of the 40 patients he examined were men, and the average age upon diagnosis was 44 (the youngest was 21 years old). Out of this, 82.5% of cases were adenocarcinomas, 12.5% were squamous cell carcinomas (SCC), and 5% were other kinds of cancer.

The bladder mucosa in untreated exstrophy was comprehensively described by Smeulders et al. in 2001 [2]. Epithelium made of transitional cells covers the trigone. The epithelium of the bladder has glandular metaplasia in the middle. Squamous metaplasia merging with healthy skin distinguishes the top epithelium from other layers. Because of the variety of epithelia lining the untreated exstrophy bladder, these individuals are more likely to develop a variety of cancers. The two most common kinds of cancer are adenocarcinomas (75%-85% of cases) and squamous cell carcinomas (5% of cases) [5,6]. Contrarily, only 0.5%-2% of bladder malignancies in the general population are caused by adenocarcinomas [2,6].

Histological subtypes of bladder adenocarcinomas might be mixed, enteric, mucinous, signet ring cell, or non-specific. In our situation, the histology proved that the cells were signet ring cells. Due to the rarity of this mutation, only one additional instance of signet ring cell cancer in an untreated exstrophy patient has been documented in the literature [7].

According to McIntosh and Worley, the exstrophy bladder experienced glandular metaplasia as a result of environmental exposure and persistent infection, likely to provide protective mucus, where the malignant transformation took place [5]. The intestinal epithelium that covers the organ's mucosa is assumed to be the source of adenocarcinoma in the exstrophy bladder. But a solid future study is required to back up these claims. The rarity of such circumstances makes a prospective study more challenging. Based on Smulders's research, the most likely scenario is that persistent irritation and infection will lead to urothelium morphing metaplastically and experiencing malignant alterations [2].

Such situations are still typically treated with surgery. Adenocarcinoma and squamous cell carcinoma are examples of non-urothelial carcinomas for whom systemic treatment has not been effective [8,9]. The recommended line of care in these circumstances, after ruling out any likely primary cancer sites, is a radical cystectomy with extensive local excision of the skin margin and ileal conduit.

Lower abdominal defect correction after exstrophy bladder excision is challenging due to the typical large abdominal defect and widely separated rectus seen in these individuals due to pubic divergence. Therefore, the alternatives for repairing the defect are the rectus abdominal rotation flap, fasciocutaneous M-plasty, and Cardiff repair with onlay mesh repair [10-12].

We preferred a pedicle of the anterolateral thigh flap based on perforators of descending branches of the lateral circumflex femoral artery.

## Conclusions

An adult seeking care for untreated exstrophy with primary bladder cancer is unusual but possible. Since the defect is so prominent at birth, nearly all patients undergo treatment soon after birth. Our patient never consulted a physician for the presence of a lower abdomen mass and presented to us with a foul-smelling discharge from the mass. Biochemical, radiological, and histopathological examinations helped in the proper evaluation of the mass.

In our case, the histopathological examination revealed signet ring cell adenocarcinoma. We suggest radical cystectomy with an ileal conduit for these patients. With a pedicle or rotation flap, the substantial lower abdominal defect that was left open can be repaired. Therefore, a satisfactory functional and cosmetic outcome can be attained in bladder exstrophy patients who present late with good surgical care.
